# Erzhi Formula Extracts Reverse Renal Injury in Diabetic Nephropathy Rats by Protecting the Renal Podocytes

**DOI:** 10.1155/2018/1741924

**Published:** 2018-08-23

**Authors:** Jun Jiang, Jiangning Yin, Xiang Liu, Huajun Wang, Guoyuan Lu

**Affiliations:** ^1^School of Pharmacy, Jiangsu University, No. 301 Xuefu Road, Zhenjiang, Jiangsu 212013, China; ^2^Affiliated Hospital of Jiangsu University, No. 438 Jiefang Road, Zhenjiang, Jiangsu 212001, China; ^3^The First Affiliated Hospital of Soochow University, No. 188 Shizi Road, Suzhou, Jiangsu, 215006, China

## Abstract

Podocytes injury was a crucial factor resulting in diabetic nephropathy (DN). Erzhi formula extract (EZF) was a clinical effective Chinese medicine on DN, but its mechanism was unclear. In this study, the main compounds of EZF and their pharmacokinetics in rat were detected by HPLC-MS/MS. And then, blood glucose, urine protein, renal index, renal microstructural (HE/PAS staining), inflammatory factors (IL-*β*, TNF-*α*, IL-6), and protein/mRNA expression related to the function of podocyte (CD2AP and Podocin) in DN rats were investigated after the oral administration of EZF. The concentrations of specnuezhenide and wedelolactone in rat kidney were 7.19 and 0.057 mg/kg, respectively. The T_max_ of specnuezhenide and wedelolactone were 2.0 and 1.50 h, respectively. Their C_max_ were, respectively, 30.24 ± 2.68 and 6.39 ± 0.05 *μ*g/L. Their AUC_(0-∞)_ were 123.30 ± 2.68 and 16.56 ± 0.98 *μ*g/L⁎h, respectively. Compared with the model group, the blood glucose and the 24-hour urinary protein were significantly decreased (P < 0.05) after 16 weeks' treatment of EZF. The expressions of Podocin and CD2AP protein/mRNA were increased (P < 0. 05). The deteriorate of glomerular morphology was alleviated under the treatment of EZF. EZF prominently decreased the levels of inflammatory factors (P < 0.05). MDA was significantly decreased (P < 0.05) with the significant increase of SOD activity (P < 0.05) in EZF groups. All the results proved that EZF repaired glomerular mesangial matrix, protected renal tubule, and improved renal function in DN rats by upregulating the expression of Podocin and CD2AP protein/mRNA in podocytes.

## 1. Introduction

In recent years, effective monomers or extracts from natural plants have been widely applied to the treatment of diabetic nephropathy (DN) [1–3]. Erzhi formula was composed of Ligustri lucidi fructus and Eclipte herba by the ratio 1:1 [4]. It can provide nutrients to the liver and kidneys and strengthen the waist and knee joints [5]. Erzhi formula extract (EZF) contained lots of active compounds such as specnuezhenide, wedelolactone, oleanolic acid, acetyl oleanolic acid, and ursolic acid [6, 7]. Among these compounds, oleanolic acid ameliorated DN and protected renal microstructural by reducing oxidative stress and endoplasmic reticulum stress in type II diabetic rats [8–11]. Ursolic acid prevented renal damage by lowering blood glucose, inhibiting the early damage of DN, suppressing oxidation and inflammatory reactions [12–14]. The specnuezhenide and wedelolactone resisted DN by inhibiting the signaling pathways related to inflammation such as HIF-1*α*/VEGF [15] and NF-kappa B [16].

DN was one of the major microvascular complications of diabetes [17]. It was characterized by accumulating glomerular extracellular matrix and tubulointerstitial compartments, thickening the intrarenal vasculature [18–20]. In recent years, the podocytes, glomerular filtration barrier structures [21], have been widely concerned. Podocytes were highly specialized cells of the renal glomerulus that wrapped around capillaries and neighbor cells of the Bowman's capsule [22–25]. Podocytes injury was extremely important in the pathogenesis of DN and was also the important target of many pathogenic factors in the process of DN [26]. When the podocytes damage was exceeded, the compensatory capacity, the filter membrane, could be destroyed which induced in large amounts of proteinuria [27–30]. Proteinuria was an important clinical marker of early DN. Therefore, the protection of podocytes was closely related to the treatment of DN. Many effective compounds and herb extracts have been discovered to protect or repair podocyte injury, such as Cordyceps sinensis, tripterygium wilfordii polyglycosidium [31], and astragalus membranaceus [32]. CD2AP and Podocin were important structural proteins of the podocyte silt membrane and maintained the glomerular filtration function and regulated proteinuria [33, 34].

The development of DN was directly linked to inflammation [35]. There were many inflammatory factors, such as IL-17A [36], IL-*β*, TNF-*α*, IL-6, MCP-1, ICAM-1, VCAM-1, IL-1, and IL-18 [37]. However, IL-*β*, TNF-*α*, and IL-6 were classical inflammatory factors for studying DN [38]. Superoxide dismutase (SOD) was an extremely important antioxidant enzyme in the human body that had multiple functions such as antiaging, immune regulation, and lipid regulation. Malondialdehyde (MDA) levels usually reflected the oxidative damage in cells and tissues [39]. In summary, these indicators were important for studying DN.

In this paper, a HPLC-MS/MS method for simultaneously detecting specnuezhenide and wedelolactone was established which was applied to the quality control and the pharmacokinetic study of EZF. And then, by replicating the DN in rats, the effects of EZF on blood glucose, proteinuria, renal index, pathological changes of renal tissue, expression of Podocin/CD2AP protein and mRNA, the IL-*β*, TNF-*α*, IL-6, and MDA levels, and SOD activity in renal tissue were evaluated. Finally, we described the mechanism of EZF in treating and reversing the DN.

## 2. Materials and Methods

### 2.1. Instruments and Reagents

Ecliptae Herbs and Ligustri Lucidi Fructus were purchased from Zhenjiang Ren Tang Pharmaceutical Co., Ltd. (Zhenjiang, China) and identified by Professor Chen Jun of Jiangsu University as* Eclipta prostrata* L. and* Ligustrum lucidum* Ait., respectively. Streptozotocin was obtained from Sigma-Aldrich (S0131, Sigma). Irbesartan was purchased from Sanofi Winthrop Industries (0.15 g/tablet, H20040494, Beijing). Glucose meter was Ouch surestep lood (ONETOUCH, Johnson China Co., Ltd.). Three triple quadrupole LC-MS/MS (Thermo TSQ Quantum) was purchased from Thermo Fisher Scientific (America) which is equipped with QED-MS/MS system. The polyclonal antibody of rabbit anti-rat Podocin was purchased from American Abcam Company (GR211434-1, USA). The polyclonal antibody of rabbit CD2AP was provided by Beijing Boosen biological (G3828, Bioss Antibodies, China); TRIzol Reagent was provided by Life Technologies (50175111, Life Technologies, USA); M-MLV reverse transcription kit was obtained from American Invitrogen Company (RP1105, Invitrogen Co., Ltd., USA). Primers were purchased from Suzhou Genewiz Technology Co., Ltd. (NTKW-20171103, Suzhou, China). High-sugar and high-fat feed were provided by Beijing Botai Hongda Biological Technology Co., Ltd. (S20160808, Beijing, China). The first strand of cDNA Synthetic kit was purchased from Vazyme Biotech Co., Ltd. (04896866001, Nanjing, China). Ethanol, chloroform, and isopropanol were analytical pure grade reagents, obtained from Sinopharm Group Co., Ltd. IL-1*β* (A1001), IL-6 (A1005), TNF-*α* (A1012), Superoxide Dismutase (SOD, A001-3), Malonaldehyde (MDA, A003-1), and urine protein assay kit (C035-2) were purchased from Nanjing built Biotechnology Co., Ltd (Nanjing, China).

### 2.2. Animals

Male SPF SD rats weighing 220 ± 20 g were originally obtained from the Animal Center of Jiangsu University. The animal certificate number was SYXK 2013-0036. The animal experiments were conducted by the Animal Ethics Committee. The rats were in-house fed separately at 23°C and 40 % humidity. Animals were maintained on standard laboratory chow and daily 12-hour light/dark cycles. All animals were provided with standard ordinary feed and water.

### 2.3. Preparation of EZF

The dried Erzhi formula was crushed and extracted with 80 % aqueous ethanol for two times (2 h for each) under heat and reflux. The extracts were dried by rotary evaporator after two extracts were combined and the ethanol was recovered. The dry powders (0.2 g/g of crude drug) were kept dry and preserved in dark place for subsequent experiments.

### 2.4. Simultaneous Determination of Main Components in EZF

#### 2.4.1. Chromatographic Conditions

The chromatographic separations were performed on Agilent ZORBAX SB-C_18_ column (150 mm × 2.1 mm, 5 *μ*m). The HPLC mobile phase which consisted of methanol (A) and 0.1% formic acid (B) was used to conduct gradient elution. The flow rate of gradient elution was 0.3 mL/min, and the column temperature was room temperature.

#### 2.4.2. Mass Spectrometry Conditions

The mass spectrometry analysis was performed with an electrospray ionization source in the negative ion detection mode, and the scanning mode was multiple reaction monitoring. Resolution was unit mass resolution. Capillary voltage and temperature were 3000 V and 350°C, respectively. Nitrogen was used as collision gas. The mass spectral parameters of the two compounds were shown in Table 1.

### 2.5. Solutions and Samples Preparation

EZF samples: 20 mg of specnuezhenide and wedelolactone were precisely weighed, respectively, and then appropriate amounts of methanol were added for ultrasonic-assisted dissolution. Then it was set to 100 mL as standard solution. The standard solution was diluted into a series of different concentrations for the establishment of standard curve. 10 mg of EZF was weighed and dissolved with 10 mL methanol by the assistant of ultrasound and then fixed up to 50 mL by methanol. The microporous filter membrane with 0.22 *μ*m was used for filtration for subsequent HPLC-MS/MS detection. All samples were operated in parallel 6 times.

Plasma sample (n = 6): EZF was suspended in 0.5 % sodium carboxymethyl cellulose and administrated orally. 0.4 mL rat blood was collected and placed in a centrifuge tube with heparin at 0.00, 0.083, 0.25, 0.5, 1, 1.5, 2, 4, 6, 8, 10, and 12 h from posterior orbital plexus, respectively. All the samples were centrifuged at 5 000 r/min for 15 min. And then, 0.1 mL supernatant was taken out and 0.2 mL acetonitrile was added to precipitate protein.

Tissue sample (n = 6): Rats were orally administrated with EZF (10 g/kg). 2 hours later, their heart, liver, spleen, lung, and kidney were taken out, washed clean, and homogenated (1.0 g/mL). 0.2 mL supernatant from homogenate liquid was taken out and 0.2 mL acetonitrile was added to precipitate protein.

All plasma and tissue samples were vortex (2 min), centrifuged (10 000 r/min, 10 min), and filtered (0.22 *μ*m) in turn followed by N_2_ blow dry. The residue was accurately added to 200 *μ*L methanol, vortexed (2 min), centrifuged (12 000 r/min, 10 min), and filtered (0.22 *μ*m) before HPLC-MS/MS analysis.

### 2.6. Establishment of Diabetic Rat Model and Grouping

After SD rats were fed for 1 week and adapted to the environment, the detection of urine protein and urine sugar was negative. A total of 48 rats were fed with a small dose of STZ, high-glucose, and high-fat diet to duplicate DN rats. All rats were fed with high-glucose and high-fat diet for 4 weeks. The rats were fasted for 12 hours and then injected 1% STZ (40 mg/kg, 0.1 mol/L citrate buffer, and pH4.5) by intraperitoneal injection. The caudal vein blood glucose was measured after 72 h, and rats with persistent hyperglycemia over 16.7 mmol/L were considered to be diabetic model and selected for further experiments [40, 41]. During the modeling process, the dead rats and the noncompliance rats were excluded. The diabetic rats were randomly divided into model group (MOD), Irbesartan (IRB, 15 mg/kg) group [42, 43], EZF high-dose (EZF-H, 15 g/kg) group, EZF middle-dose (EZF-M, 10 g/kg) group, and EZF low-dose (EZF-L, 5 g/kg) group (n=8). Rats in normal group were fed same volume of distilled water, and all groups were continuously fed for 16 weeks.

### 2.7. Related Index in the Treatment of DN

#### 2.7.1. Blood Glucose

After the intervention of the EZF, the blood glucose level of samples was measured in the second, fifth, and eighth weeks, which were taken from the tail veins of rats.

#### 2.7.2. Urine Protein and Inflammation Markers

The total of 24 hours urinary protein and the concentration of IL-*β*, IL-6 and TNF-*α* in the renal were detected according to the instructions and steps of kit. Standard solution holes and sample holes were, respectively, set on the test board, and then 50 *μ*L standard solution of different concentrations was added into the standard solution. After the sample was added into the sample hole (10 *μ*L), the diluent was added (40 *μ*L). Standard and sample holes were added with horseradish peroxidase (HRP) labelled antibody (100 *μ*L) to detect the antibody. The reaction pore was sealed with the sealing plate membrane and kept in 37°C water bath for 60 min. Discard the liquid, dry the plates, fill them up with washing fluid for 1 minutes, shake off the washing liquid, dry the plates, and repeat the washing process 5 times. The substrates were added (50 *μ*L) to all the holes and incubated for 15 minutes at 37°C. 15 minutes later, the OD value of each pore was measured at 450 nm wavelength. The standard curve was drawn with the standard concentration as abscissa and the OD as the ordinate, and the corresponding concentration of the OD value of the sample was found on the standard curve.

#### 2.7.3. Renal Index

After the rats were sacrificed, the bilateral renal tissues were taken. Then renal tissues were weighed after the renal surface blood was blotted with filter paper.

(1) Renal  index=total  bilateral  renal  weight  gbody  weight  kg×100%

#### 2.7.4. Renal Pathological Examination

The renal cortex was taken and fixed by 10 % formaldehyde for 24 hours. It was dehydrated by various concentrations of ethanol (70%, 80%, 90%, 95%, and 100%) in turn for 30 min each followed by rinsing with water, cleared in xylene and embedded in paraffin and cut into 2 *μ*m thick slice. Finally, the pathological changes of renal tissues were observed under light microscope after HE and PAS staining.

#### 2.7.5. Expression of CD2AP and Podocin Protein/mRNA

The expressions of CD2AP and Podocin protein were detected by Western blotting. After rat renal tissue 60 mg was weighed, RIPA lysis buffer was used to extract RNA and the concentrations of proteins were quantified with Bradford protein quantitation kit. 30 *μ*g sample was weight for each group. The solution was well-mixed after the total protein samples and the buffer solution of the protein gel electrophoresis were added to each group sample. The mixtures were ice bathed after denaturation for 10 min at 95°C. These samples (30 *μ*g) were slowly added into the gel hole. When the electrophoresis apparatus is under the stabilivolt state of 80 V, the samples passed through the spacer gel and the separation gel (voltage 8 V/cm). When the dye was electrophoresed to a suitable position in the separation gel, the samples were transferred to PVDF membrane on ice. The PVDF membrane was blocked with 5% skim milk powder at 4°C. The Podocin primary antibody (1:1000) and the CD2AP primary antibody (1:300) of rabbit anti-rat were added to PVDF membrane. The primary antibody membrane was added to secondary antibody solution (1:5000) after primary antibody membrane was washed with TBST. At room temperature, secondary antibody membrane was shaken slowly in dark. The membrane was washed after 60 minutes, was colored with TMB, was exposed, and was washed film. The gray ratio of the target protein/*β*-actin was used to express the relative ratio of the target protein. Specific operations are shown in the literature [44]. Primer sequences and PCR product sizes are shown in the Table 5.

### 2.8. Statistical Processing

Experimental data were expressed as average value ± standard deviation ($\overset{-}{x}\pm s$), and one-dimensional analysis of variance was performed by using GraphPad Prism 5.0 software. P < 0.05 was considered a significant difference. The DAS 2.0 software was applied to calculate the main pharmacokinetic parameters of specnuezhenide and wedelolactone, respectively.

## 3. Results

### 3.1. Chemical Composition Analysis

#### 3.1.1. Method Validation

**Figure 1:**
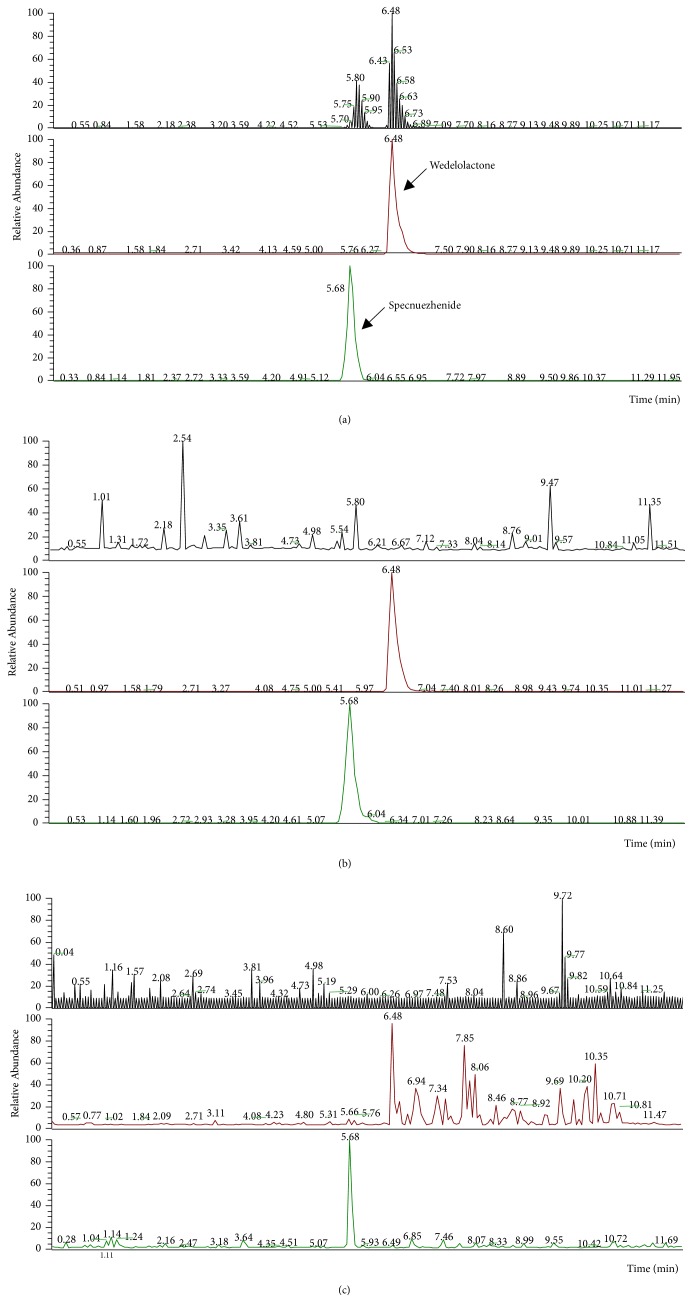
**HPLC-MS/MS chromatogram of specnuezhenide and wedelolactone in EZF, serum and tissue**. (a) The HPLC-MS/MS chromatogram of mixed standard; (b) the HPLC-MS/MS chromatogram of EZF; (c) the HPLC-MS/MS chromatogram in serum and tissue after oral administration of EZF.

*(1) Standard Curve and Linear Range*. Linear regression equation was figured through the peak area Y and concentration X (*μ*g/mL). The linear regression equations of specnuezhenide and wedelolactone were Y = 123.408 + 181.663X (R^2^ = 0.9999) and Y = 92127.2 + 3822.94X (R^2^ = 0.9987). The results showed that there was a good linearity in the range of 0.01-20 *μ*g/mL for specnuezhenide and wedelolactone. Their limit of determination (LOQ) was 0.05 *μ*g/mL. The representative HPLC-MS/MS spectrum was shown in Figures 1(a) and 1(b).

*(2) Stability and Recovery*. The abovementioned standard solution was accurately taken at 0, 1, 2, 4, and 8 hours and analyzed. The relative standard deviations of specnuezhenide and wedelolactone peak areas were 0.13 % and 1.42 %, respectively, indicating that these compounds had good stability. In addition, the known contents of 3 samples were taken and 0.5 times of standard solution were accurately added to these sample solutions. According to the preparation method of the samples, the recovery rates were calculated. The results showed that the recoveries of specnuezhenide and wedelolactone were 101.2 % and 100.3 %, indicating that they had good recovery rates.

10 *μ*L mixed standard solution (10 *μ*g/mL) was added into 0.1 mL blank plasma samples which were obtained from rat and their peak areas were measured by HPLC-MS/MS as “A” after the pretreatment. Another 10 *μ*L mixed standard solution (10 *μ*g/mL) was added into 0.1 mL methanol with the same operation according to the plasma samples and the peak areas were measured as “B.” And then calculate their recovery by “(A/B)×100%”. The recovery rate of specnuezhenide was 84.36 % and the recovery rate of wedelolactone was 86.06 %, which indicated that they all had good recovery rates.

*(3) The Content of specnuezhenide and Wedelolactone in EZF*. The content of specnuezhenide in EZF was 9.79 %, and the content of wedelolactone was 0.61 %. The representative HPLC-MS/MS spectrum was shown in Figures 1(a) and 1(b).

### 3.2. The Tissue Distribution and Pharmacokinetics of EZF in Rats

#### 3.2.1. The Tissue Distribution of Specnuezhenide and Wedelolactone

As shown in Table 2, the concentrations of specnuezhenide in the lung, renal, and liver tissues were 8.56, 7.19, and 1.52 mg/kg, respectively, after the oral administration of EZF, but the specnuezhenide in heart and spleen was not detected. The concentrations of wedelolactone in spleen, lung, liver, and renal were 5.78, 0.089, 0.057, and 0.20 mg/kg, respectively, but the wedelolactone in heart was not detected. Therefore, specnuezhenide was easily distributed in the lungs and kidney and wedelolactone was easily distributed in the spleen after the administration of EZF. The representative HPLC-MS/MS spectrum was shown in Figure 1(c).

#### 3.2.2. The Pharmacokinetics Study of EZF in Rats

**Figure 2:**
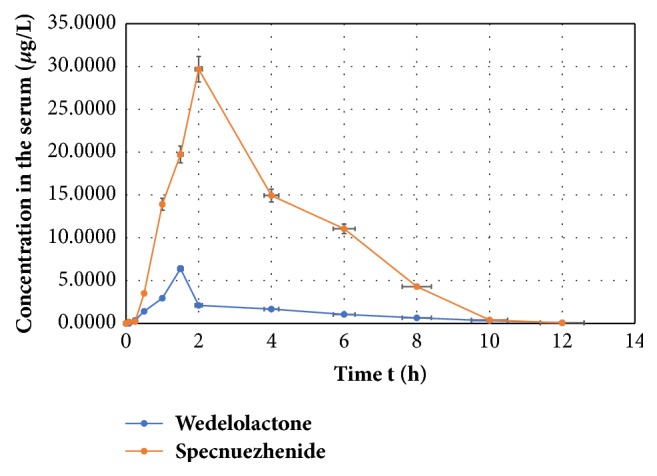
The concentration-time curve of specnuezhenide and wedelolactone in rats after oral administration of EZF ($\overset{-}{x}$, n = 6).

According to the pharmacokinetics parameters, the peak time (T_max_) of specnuezhenide and wedelolactone was 2.0 h and 1.50 h, respectively, indicating that the absorption of wedelolactone was faster than specnuezhenide. However, the C_max_ of specnuezhenide (30.24 ± 1.65 *μ*g/L) was bigger than wedelolactone (6.39 ± 0.05 *μ*g/L). In addition, the AUC_(0-*∞*)_ of specnuezhenide (123.30 ± 2.68 *μ*g/L*∗*h) was also significantly higher than wedelolactone (16.56 ± 0.98 *μ*g/L*∗*h). The results were shown in Figure 2 and Table 3.

### 3.3. Hypoglycemic Effect of EZF

**Figure 3:**
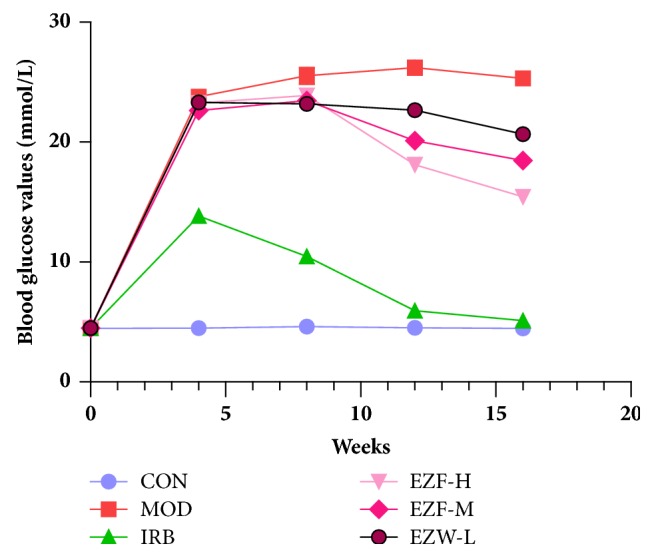
The effect of EZF on blood glucose at different time in diabetic rats ($\overset{-}{x}$, n=8).

In the normal group, the blood glucose level was maintained at 4.49 ± 0.02 mmol/L. In model, the blood glucose level gradually tends to be stable (23.78 ± 0.21 mmol/L) after 4 weeks (P < 0.01). After 16 weeks' treatment of Irbesartan, the blood glucose level reached 5.10 ± 0.20 mmol/L, which was not significantly different compared with the normal group. The blood glucose level did not decrease significantly after 4 weeks' treatment of EZF. However, the blood glucose level in DN rats decreased significantly from 8^th^ to 16^th^ week. Compared with the model, the blood glucose levels of EZF-H (15.43 ± 0.39 mmol/L), EZF-M (18.47 ± 0.15 mmol/L), and EZF-L (20.67 ± 0.49 mmol/L) were significantly decreased (P < 0.05) at the 16^th^ week. Different doses of EZF group showed dose dependence (Figure 3).

### 3.4. The Effect of Reducing Proteinuria

**Figure 4:**
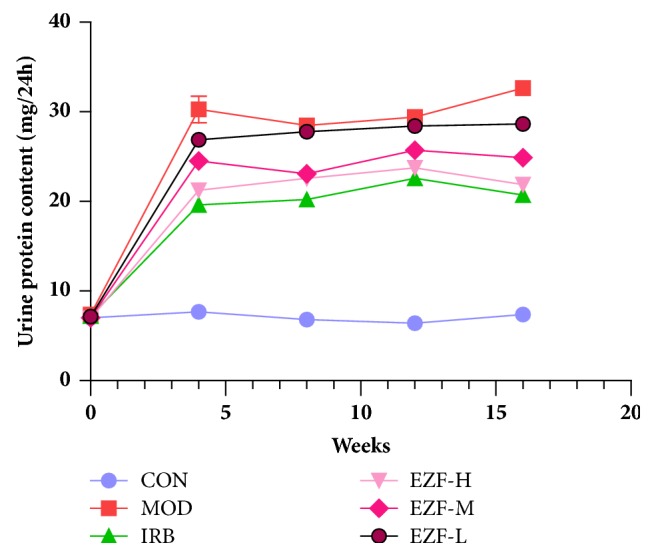
The effect of EZF on 24 hours urine protein in diabetic rats ($\overset{-}{x}$, n=8).

Compared with normal group, the 24h urinary protein level in the model group was increased significantly (P < 0.01). Compared with the model group, 24h urinary protein of Irbesartan group was decreased significantly (19.60 ± 0.24 mg, P < 0.05). The 24h urinary protein levels in EZF-H (20.72 ± 0.45 mg), EZF-M (24.88 ± 0.42 mg), and EZF-L (28.65 ± 0.46 mg) were significantly reduced (P < 0. 05). The results were shown in Figure 4.

### 3.5. Body Weight, Renal Weight, and Renal Index Changes

Compared with the normal group, the body weight of model group was decreased significantly (P < 0.01); renal weight and renal index increased significantly (P < 0.01). Compared with the model group, Irbesartan significantly reversed the body weight and renal weight of the model group (P < 0.01) and the renal index decreased significantly (P < 0.01). After the intervention of the EZF, EZF-H and EZF-M groups significantly reversed the body weight, kidney weight, and renal index of diabetic rats (P < 0.05). The specific results are shown in Table 4.

### 3.6. Inflammatory Factors and Antioxidant Markers in the Renal Injury

**Figure 5:**
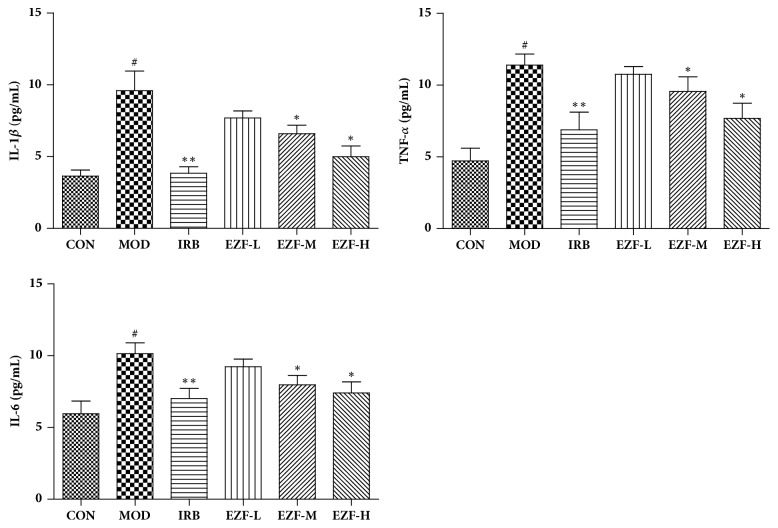
**The effect of EZF on renal inflammation markers in diabetic rats (** $\overset{-}{x}$ **, n=8)**. CON: blank control group. MOD: model group. IRB: Irbesartan group. EZF-L: Erzhi formula low-dose group. EZF-M: Erzhi formula middle-dose group. EZF-H: Erzhi formula high-dose group.* Note*. #: compared with normal group, P < 0.01. *∗∗*: compared with model group, P < 0.01. *∗*: compared with model group, P < 0.05. Statistical analysis was completed by GraphPad Prism 5.0 software.

Compared with the normal group, IL-*β*, TNF-*α* and IL-6 in the renal podocytes of model were significantly increased (P < 0.01). The Irbesartan decreased these inflammatory factors in the model group (P < 0.01). Compared with the model group, the EZF-H and EZF-M significantly reduced IL-*β*, TNF-*α*, and IL-6 (P < 0.05). The results are shown in Figure 5.

**Figure 6:**
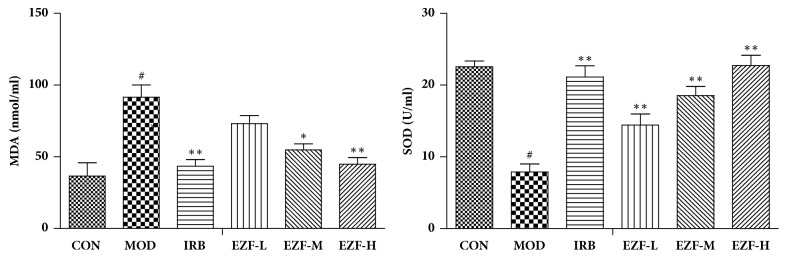
**SOD activity and MDA concentration in renal of each group (** $\overset{-}{x}$ **, n=8)**.* Note*. #: compared with normal group, P < 0.01. *∗∗*: compared with model group, P < 0.01. *∗*: compared with model group, P < 0.05. Statistical analysis was completed by GraphPad Prism 5.0 software.

The results of antioxidant assays showed that SOD activity in the renal of the model group decreased significantly (P < 0.01) and the MDA concentration increased significantly (P < 0.01). Compared with the model group, the MDA in rats renal were significantly reduced under the treatment of EZF-H and EZF-M (P < 0.05). Furthermore, all EZF groups significantly increased SOD activity (p< 0.05). The results are shown in Figure 6.

### 3.7. HE and PAS Staining

**Figure 7:**
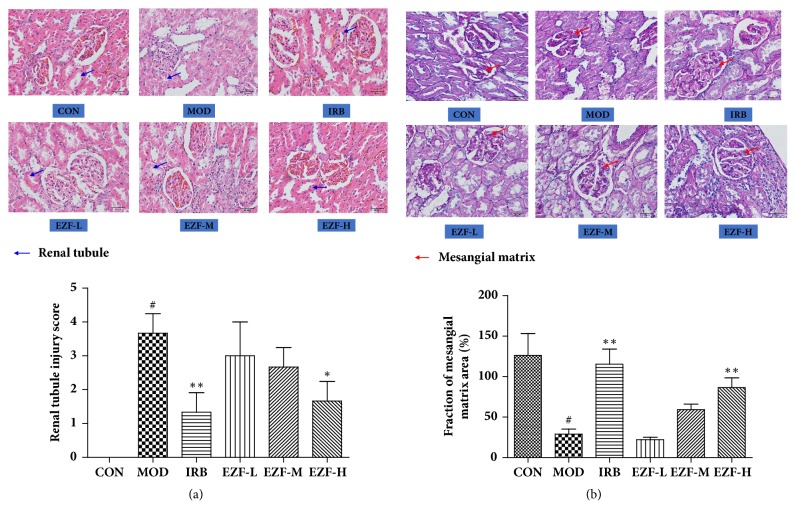
**HE and PAS staining of renal pathological changes in rats**. (a) HE staining of renal tubule pathological changes (× 400). (b) PAS staining of glomerular mesangial matrix pathological changes (× 400). The fraction of mesangial matrix area = (mesangial matrix area/glomerular area) × 100%. The rules of renal tubule injury score were as follows: 0 points, normal kidney; 1 points, minimal necrosis (< 5% renal tubular necrosis); 2 points, mild necrosis (5%-25% renal tubular necrosis); 3 points, moderate necrosis (25%-75% renal tubular necrosis); 4 points, severe necrosis.* Note*. #: compared with normal group, P < 0.01. *∗∗*: compared with model group, P < 0.01. *∗*: compared with model group, P < 0.05. Statistical analysis was completed by GraphPad Prism 5.0 software.

In the Normal group, the kidney had normal renal morphology, clear structure, regular globules, no expansion of capillary lumens, tight arrangement of tubules, normal renal tubular epithelial cell morphology, no glomerular atrophy or hypertrophy, no thickening of the basement membrane, and no mesangial proliferation. Compared with the normal group, the glomerular volume of the model group rats increased and the capillary basement membrane thickened significantly. The proliferation of mesangial matrix, vacuolar degeneration of renal tubular epithelial cells, and protein tube type can be observed in the model group. EZF and the Irbesartan groups alleviated the pathological changes of DN rats. The glomerular morphology was basically normal. The thickened basement membrane and the hyperplasia of mild mesangial were obviously observed. There was a small amount of vacuolar degeneration in renal tubular epithelial cells and the lesion extent was significantly lighter than the model group. The HE and PAS staining, the fraction of mesangial matrix area, and renal tubule injury score were shown in Figure 7 (Figures 7(a) and 7(b)).

### 3.8. CD2AP and Podocin Protein/Gene Expression

**Figure 8:**
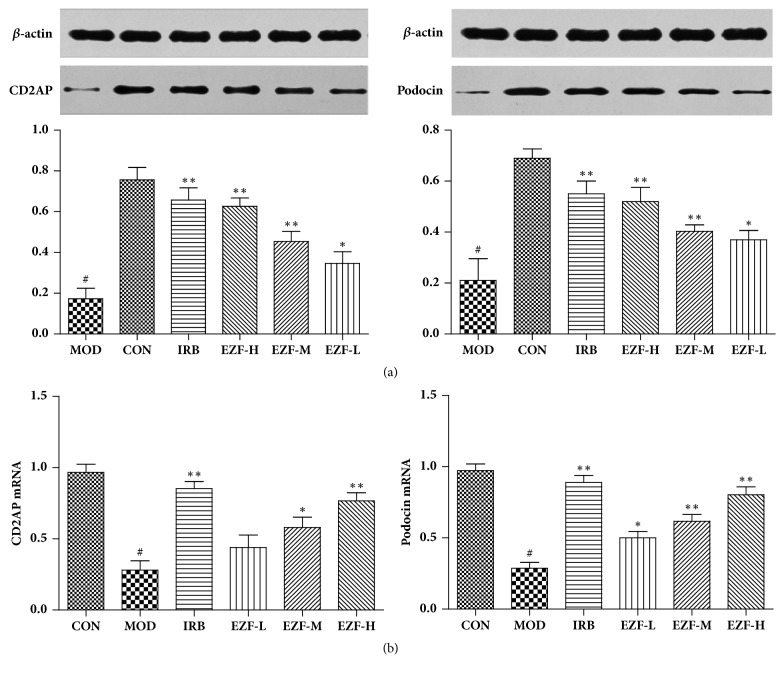
**Podocin and CD2AP protein/gene expression in rats renal of each group (** $\overset{-}{x}$ **, n=8)**. (a) Podocin and CD2AP protein expression. (b) Podocin and CD2AP gene expression.* Note*. #: compared with Normal group, P < 0.01. *∗∗*: compared with Model group, P < 0.01. *∗*: compared with Model group, P < 0.05. Statistical analysis was completed by GraphPad Prism 5.0 software.

Compared with the normal group, the Podocin and CD2AP protein/gene in the renal tissue of the model group were decreased (P < 0. 01). Compared with the model group, the expression of Podocin and CD2AP protein/gene in the IRB, three EZF groups, were increased (P < 0. 05). The protein and gene expression of Podocin and CD2AP were shown in Figures 8(a) and 8(b), respectively.

## 4. Discussion

So far, HPLC was mainly applied for the quality control of Erzhi formula. Although this method satisfied the determination of the main components in medicinal materials, its sensitivity and accuracy cannot satisfy the needs of biological samples such as serum and tissues. Therefore, the HPLC-MS/MS method was established for the first time in simultaneous determination of wedelolactone and specnuezhenide which derived from Eclipte herba and Ligustri lucidi fructus respectively. Our method was more scientific and efficient to control Erzhi formula quality and to avoid false positive results through accurately quantifying wedelolactone and specnuezhenide. Moreover, this method was beneficial for the pharmacokinetics study of EZF.

The production of proteinuria was mainly related to glomerular filtration barrier. Slit diaphragm (SD) was the final barrier to glomerular filtration and acted as an important role in the development of proteinuria [45]. Studies showed that the nephrin/CD2AP/Podocin complex was necessary to maintain the glomerular filtration function [46]. Podocin was the key functional unit of SD, which had ion channels and signal transduction functions and maintained the structure and function of SD [47]. Podocin also interacted with the intracellular segment of CD2AP and exerted the effect of functional complex by forming lipid raft-like structures [48]. Podocin connected the tight junction protein with the actin of podocyte to exert a scaffolding function which was concentrated on tight junctions of foot processes [49]. Podocin's multiple mutations (V180M, R238S, G92C, etc.) interfered with the structural connectivity of nephron, leading to podocyte pathological changes and massive proteinuria [50]. CD2AP was a transmembrane protein belonging to the immunoglobulin superfamily. Its structure indicated that CD2AP regulated the cytoskeleton and also mediated the interaction of related proteins [51]. When CD2AP expression was decreased, it induced cytoskeletal disruption and podocyte apoptosis [45]. CD2AP had an important role in promoting the binding of T cell surface antigen CD2 to antigen-presenting cells [52]. When the function of CD2AP was abnormal, antigen recognition and presentation were not able to be performed which resulted in overimmunization, T-cell death, and proteinuria. Studies reported that the mice of deleting CD2AP gene caused defects in the renal podocyte foot processes, produce large amounts of proteinuria. It also increased the susceptibility of glomerular injury, leading to glomerular sclerosis. Defects in CD2AP caused fusion of the foot processes through deposition of the mesangial matrix, resulting in proteinuria [53–56].

In recent years, studies indicate that DN was an inflammation-related disease [36]. DN rats produced a variety of inflammatory factors under a high-sugar environment. Inflammatory factors acted on the renal through different pathways to accelerate the development of DN [57]. Therefore, the inflammatory response was an important research direction of DN and was the development of DN had wide concern, which was an important research direction of DN [58]. TNF-*α*, an inflammatory factor through the NF-*κ*B signaling pathway, activated a variety of inflammatory factors. Moreover, permeability of endothelial cells and production of proteinuria were increased due to TNF-а accumulation and attachment to the glomerular endothelium [59]. IL-6 induced insulin resistance and insulin secretion dysfunction by promoting lipid oxidation and participates in promoting the occurrence and development of DN [37, 60].

This experiment successfully replicated DN rats. The results showed that EZF effectively reduced the urinary protein, significantly decreased the IL-*β*, TNF-*α*, IL-6, and MDA, increased SOD activity, and unregulated the expression of CD2AP, Podocin proteins, and mRNA in renal silt membrane. The renal protective effect of EZF on DN rats was related to upregulate Podocin and CD2AP mRNA/protein expression and reduced renal injury by inhibiting inflammatory reaction. In summary, EZF reversed renal injury in diabetic nephropathy rats by protecting the podocytes and inhibiting the renal inflammation.

High blood glucose level causes the damage of glomerular and podocyte [61]. In this paper, the positive drug, Irbesartan, was not a hypoglycemic drug, but it showed the hypoglycemic effect which deserves further study. In addition, EZF was also not a hypoglycemic drug and its regulation of blood glucose was very weak (Figure 3). However, EZF exhibited a better role in reducing proteinuria, inhibiting renal inflammation and antirenal oxidation, improving glomerular function, and increasing podocyte functional protein. There was no significant difference between Irbesartan and EZF (EZF-H) in improving diabetic nephropathy. These data proved that EZF had a good antidiabetic nephropathy effect. Pharmacokinetic data also showed that the main components of EZF, especially specnuezhenide and Wedelolactone (Table 2), could reach renal tissue and the distribution concentration of specnuezhenide was high. Therefore, we speculated that specnuezhenide was an important substance of EZF in antidiabetic nephropathy. Subsequent studies will explore the molecular mechanism of the antidiabetic nephropathy of specnuezhenide.

## 5. Conclusion

EZF inhibited renal injury and repaired renal function in diabetic rats, which were mediated by protecting the podocytes and upregulating the expression of CD2AP and Podocin.

## Figures and Tables

**Table 1:** The mass spectral parameters of specnuezhenide and wedelolactone.

Chemical compound	Parent ion (m/z)	daughter ion (m/z)	Residing time (ms)	Cracking voltage (V)	Collision energy (V)
Wedelolactone	313	298.1^*∗*^	100	81	23
		186.0	100	81	38
Specnuezhenide	685.21	523.3^*∗*^	100	89	21
		299.1	100	89	27

^*∗*^Quantitative ion.

**Table 2:** The distribution of specnuezhenide and wedelolactone in rat tissues ($\overset{-}{x}$, n = 6).

**Tissues**	**Components (mg/kg)**	
	**Specnuezhenide**	**Wedelolactone**
Spleen	/	5.78
Lung	8.56	0.089
Heart	/	/
Liver	1.52	0.20
Renal	7.19	0.057

**Table 3:** The specnuezhenide and wedelolactone pharmacokinetic parameters of EZF in rats by intragastric administration ($\overset{-}{x}\pm SD$, n = 6).

Statistical moment parameter	Unit	Wedelolactone	Specnuezhenide
AUC (0-t)	*μ*g/L*∗*h	13.77 ± 1.17	116.60 ± 14.15
AUC (0-∞)	*μ*g/L*∗*h	16.56 ± 0.98	123.30 ± 2.68
AUMC(0-t)		51.09 ± 2.89	419.01 ± 9.19
AUMC (0-*∞*)		69.13 ± 5.51	437.67 ± 13.92
MRT (0-t)	h	3.12 ± 0.35	3.63 ± 0.08
MRT (0-*∞*)	h	4.52 ± 0.43	3.52 ± 0.22
VRT (0-t)	h∧2	5.77 ± 0.42	4.42 ± 0.05
VRT (0-*∞*)	h∧2	14.48 ± 1.01	5.55 ± 0.38
t1/2z	h	2.95 ± 0.40	2.36 ± 0.28
T_max_	h	1.50 ± 0.00	2.00 ± 0.00
C_max_	*μ*g/L	6.39 ± 0.05	30.24 ± 1.65

**Table 4:** Effect of EZF on body weight, renal weight, and renal index in diabetic rats ($\overset{-}{x}\pm SD$, n = 8).

Groups	Body weight (g)	renal weight (g)	renal Index (×100)
Normal	710.32 ± 11.54	1.57 ± 0.04	0.22 ± 0.004
Model	327.83 ± 4.83^#^	1.74 ± 0.05^#^	0.53 ± 0.020^#^
Irbesartan	376.37 ± 6.77^*∗∗*^	1.58 ± 0.04^*∗∗*^	0.42 ± 0.013^*∗∗*^
EZF-H	361.63 ± 4.30^*∗∗*^	1.59 ± 0.05^*∗∗*^	0.44 ± 0.019^*∗∗*^
EZF-M	345.70 ± 3.46^*∗*^	1.65 ± 0.02^*∗*^	0.48 ± 0.004^*∗∗*^
EZF-L	334.60 ± 1.59	1.68 ± 0.05	0.50 ± 0.012

*Note*. ^#^: compared with Normal group, P < 0.01; ^*∗∗*^: compared with Model group, P < 0.01; ^*∗*^, compared with Model group, P < 0.05. Statistical analysis was completed by GraphPad Prism 5.0 software.

**Table 5:** List of primer sequences and PCR product sizes.

Name	Sequence	Length
podocin-F_Rat	GGTTCTGCATAAAGGTTGTTCAAGA	169
podocin-R_Rat	TCATGGAAAGGTATTTCCAAGGTCT	
CD2AP-F_Rat	AGCTTCCTCAGAGAACTTGTTACAT	204
CD2AP-R_Rat	GAAAGAGATGGCTTTGAAGAGTAGC	
R_GAPDH_F266	GTGCTGAGTATGTCGTGGAGTC	175
R_GAPDH_R440	TTGCTGACAATCTTGAGGGA	

## Data Availability

The authors agree that others are free to use all the data in the article under reasonable circumstances.
